# Wild Boar and Red Deer as Key Players in Coxiella Burnetii Circulation in the Wildlife–Livestock–Human Interface: Serological Analysis Using a Gaussian Mixture Model

**DOI:** 10.3390/ani16182876

**Published:** 2026-09-12

**Authors:** Luis Barros, Sofia Anastácio, Ricardo Cabeças, Ana Carolina Abrantes, Gilberto Fernandes, Andreia Gil, Catarina Coelho, Madalena Vieira-Pinto

**Affiliations:** 1 Department of the Environment for the Climate Change, Hygiene Service, Tondela City Hall, Largo da República nº16, 3460-532 Tondela, Portugal; 2 Vasco da Gama Research Centre (CIVG), Department of Veterinary Sciences, Vasco da Gama University School, Avenida José R. Sousa Fernandes 197 Lordemão, 3020-210 Coimbra, Portugal; sofia.anastacio@gmail.com (S.A.); ricardo.cabecas@euvg.pt (R.C.); 3 Center of Neurosciences and Cell Biology, Health Science Campus, Azinhaga de Santa Comba, 3000-548 Coimbra, Portugal; 4 Animal and Veterinary Research Center, University de Trás-os-Montes e Alto Douro (UTAD), 5001-801 Vila Real, Portugal; carolina.psca@gmail.com; 5 Veterinary Sciences Department, Évora University, 7004-516 Évora, Portugal; 6 Livestock Producers Organization for Animal Health Defense in the Municipality of Vinhais, Largo Toural, 5320-311 Vinhais, Portugal; 7 Northern Regional Coordination and Development Commission-Licensing Division, Rua Doutor João Leonardo nº 5. Edifíco MAP, 5160-280 Torre de Moncorvo, Portugal; 8 Research Center for Natural Resources, Environment and Society (CERNAS), Polytechnic Institute of Viseu, Campus Politécnico, Repeses, 3504-510 Viseu, Portugal; 9 Agrarian School of Viseu, Polytechnic Institute of Viseu, 3504-510 Viseu, Portugal; 10 Associate Laboratory for Animal and Veterinary Sciences (AL4AnimalS), 5001-801 Vila Real, Portugal

**Keywords:** *Coxiella burnetii*, Q fever, wild boar, red deer, one health, wildlife

## Abstract

*Coxiella burnetii*, the etiological agent of Q fever, is a globally distributed zoonotic pathogen and a One Health priority. Despite increasing recognition of wildlife as potential reservoirs, data on *C. burnetii* circulation in European wild ungulates remain scarce. This study estimated exposure to *C. burnetii* in free-ranging wild boar (*Sus scrofa*) and red deer (*Cervus elaphus*) from two major hunting regions in east-central and northeastern Portugal and evaluated the performance of a commercial multi-species ELISA in these hosts. Between 2011 and 2021, 279 serum samples (162 wild boar; 117 red deer) from legally hunted animals in Portugal were tested using an indirect multi-species ELISA using an empirical species-specific threshold derived from a Gaussian mixture model. Overall seroprevalence was 6.8% (CI 95%; 3.4–11.8) in wild boar and 13.7% (CI 95%; 8.0–21.3) in red deer, with higher exposure in wild boar from Beira Baixa (east-central Portugal) than from Trás-os-Montes (northeastern Portugal). The manufacturer’s ruminant cut-off classified almost no animals as seropositive (0.6% and 2.6%, respectively) and showed slight to fair agreement with the species-specific thresholds, underscoring the need for validated, wildlife-appropriate diagnostic criteria. These findings confirm exposure to *C. burnetii* in both species, suggesting their possible involvement in local maintenance of the pathogen at the wildlife–livestock–human interface. The results reinforce the value of integrating wildlife surveillance into national Q fever surveillance and control strategies to safeguard both public health and livestock production within a One Health context.

## Simple Summary

Q fever is a zoonotic disease whose epidemiology remains insufficiently characterized in Portugal, particularly regarding its circulation in wildlife species, such as game animals, that are in close proximity to humans. This study assessed exposure to *Coxiella burnetii* in wild boar (*Sus scrofa*) and red deer (*Cervus elaphus*) from east-central and northeastern Portugal, using blood samples collected from legally hunted animals and a serological assay that detects evidence of prior exposure to the bacterium. Antibodies were found as evidence of exposure in both species, with notably higher exposure levels in wild boar from one of the surveyed regions. These findings indicate that free-ranging ungulates are exposed to *C. burnetii* and may take part in its local circulation, particularly where wildlife, livestock and human activities overlap. The study also showed that diagnostic assays developed for domestic ruminants may not perform optimally in wildlife, highlighting the need for assay adaptation and validation to improve surveillance. Overall, the findings underscore the importance of integrating wildlife into national monitoring and control strategies for Q fever, with the dual objective of safeguarding public health and supporting livestock health management.

## 1. Introduction

*Coxiella burnetii* is a Gram-negative obligate intracellular bacterium, which is the etiologic agent of Q Fever [1]. Q fever was first discovered in 1935, when Edward Holbrook Derrick investigated a disease among abattoir workers in Brisbane, Australia [2]. This zoonotic disease can infect many hosts, including humans, domestic animals, wildlife, and arthropods [3,4,5].

*Coxiella burnetii* displays exceptional environmental resilience, persisting for extended periods under conditions of low humidity and high temperature [6], a trait attributable to its ability to generate small-cell variants that are highly resistant to ultraviolet (UV) radiation, heat, and many disinfectants [7]. For that reason, at ambient temperature, *C. burnetii* can remain viable in soil or dust [8]. Transmission of *C. burnetii* occurs predominantly through the aerogenic route, following inhalation of aerosols or dust contaminated with the bacterium. The pathogen can adhere to dust particles, facilitating long-range dispersal and maintaining viability despite adverse environmental conditions [9]. These characteristics contribute to its wide epidemiological distribution, enabling transmission beyond direct animal-to-human contact and facilitating community-level outbreaks [10,11]. Its primary reservoirs are domestic ruminants, sheep, goats, and cattle that shed the pathogen in various bodily fluids, including milk, urine, faeces, and, most notably, birth products such as placental tissues and fetal fluid [12]. Wild boar and red deer are described here as species with serological evidence of exposure, whose reservoir competence remains to be established.

Nonetheless, inhalation of aerosols and contaminated dust from infected animals remains the main route of transmission to both animals and humans [13].

Across the European Union, 719 confirmed human Q fever cases were reported in 2022 and 805 in 2023. This represents a marked increase of approximately 56.5% in 2022 compared with the previous year, followed by a further rise of 11.5% in 2023 relative to 2022. In 2023, Portugal recorded 33 confirmed cases and a notification rate of 0.32 cases per 100,000 population, the highest number since 2019 [14]. The incidence of Q fever, a human zoonotic infection, reflects the circulation of the bacteria in the animal reservoirs, i.e., domestic ruminants and several wildlife species [15]. Despite well-documented outbreaks in humans, this disease remains underreported and under-researched in many parts of the world, including Portugal [16,17]. As a public health concern, it is essential to understand this disease well and respond to it as quickly as possible, underscoring the need for a One Health, multidisciplinary approach [17,18].

Q fever has been shown to be a significant zoonotic disease worldwide, affecting both public and animal health [19]. Within the One Health Surveillance (OHS) framework, EFSA identified a decrease in 2024 in the number of ruminant samples submitted by EU Member States compared with previous years; notably, Portugal submitted no data over the last five years [20]. Notwithstanding, across Europe, *C. burnetii*-positive results have been reported in cattle (Bulgaria, Poland, the Netherlands, Hungary, and Austria) and sheep (Austria, Belgium, Hungary, Ireland, Italy, Spain, and Switzerland) [20]. Wildlife species, together with their associated ticks, may play a role in the maintenance and transmission of *C. burnetii* [21]. Although it is biologically plausible that ticks function as both reservoirs and vectors, facilitating pathogen circulation within wildlife populations, this hypothesis remains to be fully confirmed. *C. burnetii* DNA has been detected in several wildlife-associated tick species, which may contribute to environmental dissemination [22]. Nonetheless, the detection of *C. burnetii* in ticks should be distinguished from demonstrated vector-mediated transmission. The absence of a consistent association in some studies highlights the need for further research to clarify the epidemiological significance of wildlife–tick cycles in the ecology of *C. burnetii* [23,24].

The detection of *Coxiella burnetii* DNA in tissues and serological evidence of exposure in wild game species, including red deer and wild boar, suggest that these hosts may participate in its transmission dynamics; but this does not, by itself, demonstrate that they maintain independent transmission cycles [25]. Previous studies have reported serological evidence of exposure in red deer and wild boar in east-central Portugal, suggesting that these species may be involved in local maintenance of the pathogen and could increase the risk of human and livestock infection where they coexist closely [18,21]. Wildlife may contribute to the ecological persistence of *C. burnetii* locally and, under some conditions, increase opportunities for spillover, particularly where wildlife and agricultural or peri-urban environments intersect. Environmental disturbances—such as deforestation, land-use change, and habitat fragmentation—may further intensify these interactions by elevating contact rates and thereby enhancing opportunities for pathogen transmission [22,26,27]. Additional pressures, including the gradual expansion of intensive livestock production in developed countries, perceived as a more sustainable production model, may also increase the risk of exposure at the wildlife–livestock interface [28].

Furthermore, in the Iberian Peninsula, populations of wild boar and red deer have undergone substantial and sustained growth, particularly in Portugal [19]. High densities of wild ungulates can sustain larger tick populations and have been hypothesized to intensify circulation, although a direct causal link to *C. burnetii* transmission remains to be established. Despite accumulating evidence that certain wild species may act as reservoirs of this pathogen, the influence of host- and environment-related factors remains poorly understood [29,30]. This demographic expansion reinforces the need to better understand the epidemiological role of these species in Q fever ecology.

From a public health perspective, hunters and others who handle wildlife carcasses may reduce their risk of exposure by adopting personal protective measures, such as wearing face masks, using protective clothing to limit tick contact, and avoiding consumption of raw or undercooked meat. Nevertheless, indirect transmission through aerosolized particles remains difficult to prevent, underscoring the importance of awareness, especially among individuals with underlying health vulnerabilities [21].

*Coxiella burnetii* is an obligate intracellular bacterium; its investigation in wildlife relies on direct or indirect diagnostic approaches. Among the available methods, molecular techniques—PCR/qPCR—are currently considered the most suitable for detecting *C. burnetii* DNA in active shedding or tissue carriage [31]. For epidemiological investigations in wildlife populations, however, serological assays such as ELISA are often preferred, as they enable assessment of exposure at the population level rather than confirmation of active infection [32]. However, currently available commercial ELISA kits are specifically developed and validated for domestic ruminants and are not optimized for wildlife species. When applying indirect ELISA to wildlife, two recurrent challenges were identified: the absence of species-specific secondary antibodies, and the lack of well-characterized positive and negative reference sera. Both of these points limit the robust validation and performance evaluation of the assays used. Gaussian Mixture Models (GMMs) are especially well suited to the analysis of serological data from wildlife populations in the absence of validated diagnostic cut-offs—a common limitation in wildlife epidemiology that hinders reliable classification of individuals as seronegative or seropositive using conventional cut-offs transferred from domestic ruminants, which may misclassify a variable proportion of samples. By offering a data-driven, probabilistic framework, GMMs enable differentiation between seronegative and seropositive animals without dependence on arbitrary cut-off values. As a result, GMMs constitute a statistically robust and interpretable tool for serological surveillance in wildlife, particularly when gold-standard assay validation is unavailable and traditional dichotomous thresholds risk compromising diagnostic accuracy [33,34]. These constraints highlight the need to develop and validate ELISA protocols that are suitable for use across multiple host species, including free-ranging wildlife.

The two main objectives of this study included: (1) to determine the serological exposure of free-ranging wild boar and red deer to *Coxiella burnetii* in two major hunting regions of Portugal, thereby filling a critical knowledge gap on Q fever circulation in European wildlife; and (2) to explore the performance and suitability of a commercial multi-species ELISA for detecting *C. burnetii* antibodies in wild ungulates.

## 2. Materials and Methods

### 2.1. Study Area

**Figure 1:**
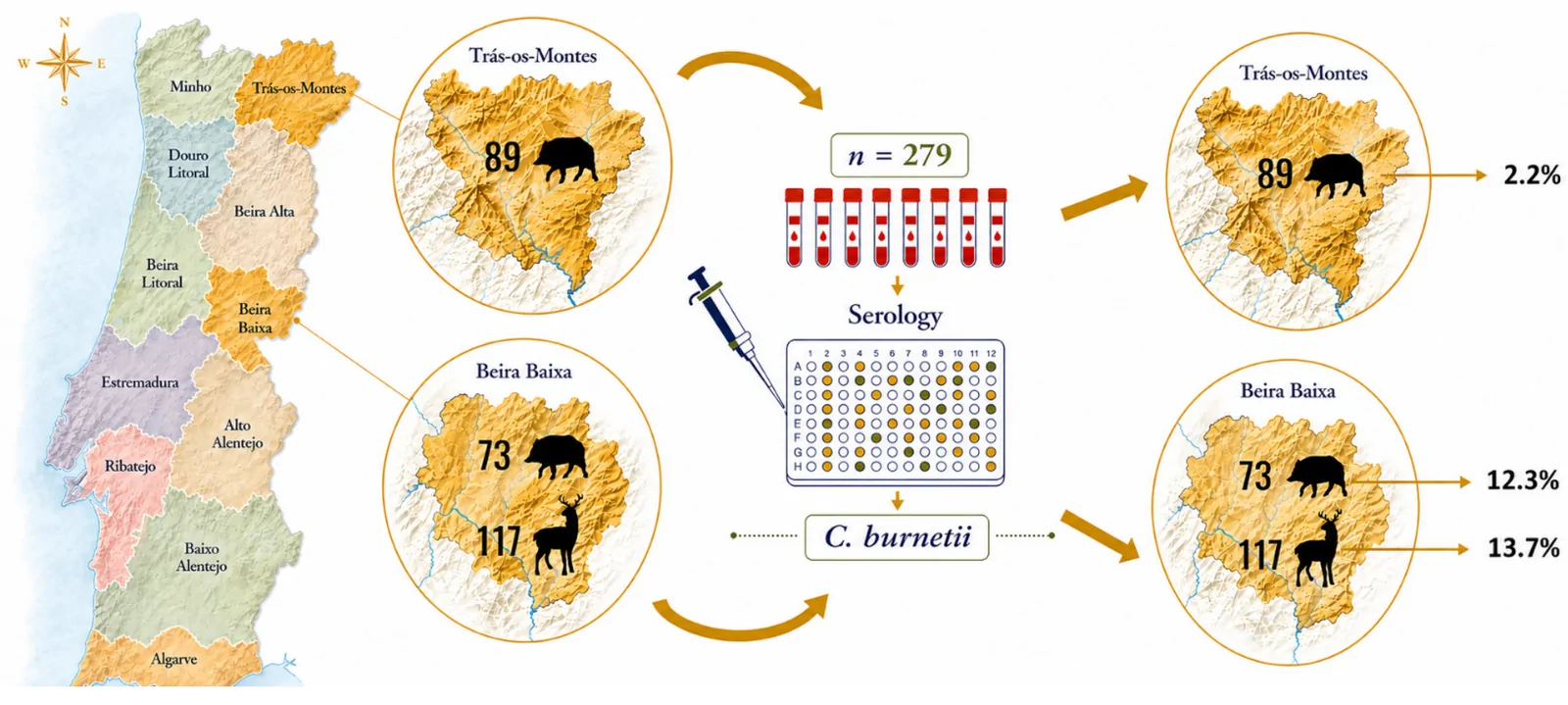
Map of Portugal with the 2 different sampling locations in focus: Trás-os-Montes and Beira Baixa, showing the exposure of wild boar and red deer in each region, defined based on the estimates generated by the Gaussian mixture model.

This cross-sectional study assessed exposure to *C. burnetii* in 162 free-ranging wild boar (*Sus scrofa*) and 117 red deer (*Cervus elaphus*) from two Portuguese regions recognized as key large-game hunting areas: Trás-os-Montes (northeastern Portugal) and Beira Baixa (east-central Portugal). Wild boar were sampled in both regions (89 in Trás-os-Montes and 73 in Beira Baixa), whereas red deer (*n* = 117) were sampled exclusively in Beira Baixa (Figure 1).

In the low-population, semi-rural Trás-os-Montes region of northeastern Portugal, hunting is a popular activity within communal and associative organizations. The most prevalent large game species in this area is the wild boar [35]. Because this region offers the most favorable circumstances for the survival of wild boar, with great food diversity, mainly fruit crops, due to the presence of large vineyards and orchards, game management is less intensive, and there is less pronounced interaction at the livestock-wildlife interface [36].

Beira Baixa is the region with the strongest hunting tradition in Portugal. Its climate, agricultural landscape, and game-management practices create highly favorable conditions for large-game species, and the area contains more than 200 officially approved hunting grounds, including associative, municipal, and tourism-managed units [37].

The region supports a diverse assemblage of large-game species, including wild boar, red deer (*Cervus elaphus*), fallow deer (*Dama dama*), roe deer (*Capreolus capreolus*), and mouflon (*Ovis aries musimon*). Among these, wild boar and red deer are the most abundant and widely harvested species [38]. Compared with Trás-os-Montes, Beira Baixa is characterized by more intensive game-management practices and a more pronounced livestock–wildlife–human interface, conditions that may facilitate pathogen circulation and increase opportunities for cross-species transmission.

### 2.2. Sampling and Laboratory Analysis

Blood samples for this study were obtained directly from the thoracic cavity upon evisceration, allowed to clot at environmental temperature, and kept refrigerated (5° ± 3 °C) until centrifugation. All collections were carried out by veterinary technical teams highly trained in post-mortem sample collection procedures. The samples were delivered to the laboratory 24–48 h after collection and subsequently centrifuged at 1500× *g* for 10 min. Serum samples were transferred into individual labelled microcentrifuge tubes and stored at −20 °C until analysis. Samples showing marked hemolysis were excluded from the study.

No animals were killed for the purpose of this study. All samples were obtained from animals legally hunted during regular seasons under applicable Portuguese wildlife-management regulations, and the study did not influence hunting decisions. Ethical approval was therefore not required; only prior explanation was required for the presence of veterinary teams at the collection/evisceration spots following driven hunts.

Each serum was tested for the presence of antibodies to *C. burnetii* using the ID Screen® Q Fever Indirect Multi-Species ELISA kit (IDvet, Montpellier, France).

This commercial indirect ELISA is based on whole-cell antigens of *C. burnetii* (phases I and II) derived from domestic ruminants and incorporates a multi-species, horseradish peroxidase-conjugated anti-immunoglobulin G (IgG) secondary antibody supplied by the manufacturer. The manufacturer validates the multi-species conjugate for use with serum from a broad range of mammalian hosts and demonstrates reactivity with IgG from multiple artiodactyl species, owing to the high degree of conservation of the immunoglobulin Fc region across mammals. However, manufacturer multi-species labeling should not be interpreted as validation for wild boar or red deer, as interspecies differences in IgG concentration and anti-IgG conjugate affinity can alter OD and S/P% values.

The ID Screen® Q Fever Indirect Multi-species kit protocol specifies testing of field samples in a single well per sample.

The PC and NC sera supplied by the manufacturer have been validated for use in cattle, sheep and goats. In the absence of validated species-specific reference sera for wild boar and red deer, these heterologous controls were used exclusively for technical validation—confirming adequate plate development, absence of non-specific background, and consistency of kit performance across runs—and not for the derivation of species-specific cut-off values.

For each sample, the sample-to-positive percentage ratio (S/P%) was calculated using the OD values of the PC and NC controls from the corresponding plate, according to the formula:

S/P% = [(ODsample − ODNC)/(ODPC − ODNC)] × 100

To define the cut-off values, two complementary approaches were used to classify samples as seropositive or seronegative.

Approach 1—Manufacturer’s cut-off: As a reference for comparability with the published literature, the manufacturer’s recommended cut-off for ruminants was applied: samples with S/P% > 50% were classified as seropositive, samples with S/P% between 40% and 50% as inconclusive (grey zone), and samples with S/P% < 40% as seronegative (ID Screen® Q Fever Indirect Multi-Species kit, Innovative Diagnostic (IDvet), Grabels, France). This cut-off was applied using the per-plate S/P% values described above.

Approach 2—Empirical species-specific cut-off: To derive a cut-off appropriate for each species, the frequency distribution of S/P% values was modeled as a two-component Gaussian mixture, under the assumption that the sampled population comprises a mixture of seronegative and seropositive subpopulations [39].

Assuming the sampled population comprised both seronegative and seropositive individuals, the distribution of S/P% values was modeled as a two-component Gaussian mixture representing these two underlying subpopulations. The model parameters were estimated using the Expectation–Maximization (EM) algorithm implemented in R (normalmixEM function, mixtools package). The fitted model was evaluated to determine whether two distinct underlying distributions adequately described the observed data, and the species-specific cut-off was derived from it. Full details of the mixture model, the cut-off derivation criterion, and the estimated model parameters for each species are provided in the Supplementary Materials (Text S1 and Tables S1 and S2).

Fisher’s exact test was employed to analyze the associations between the serological results from both regions where wild boar was present. Variables with a *p*-value < 0.05 were considered statistically significant. Agreement between the two classification methods was assessed by Cohen’s kappa coefficient (κ) with 95% confidence intervals, interpreted according to [40]. Seroprevalence estimates are presented with 95% exact binomial confidence intervals.

## 3. Results

Between 2011 and 2021, a total of 279 blood samples were collected in the northeastern (Trás-os-Montes) and east-central (Beira Baixa) regions of Portugal: 162 from wild boar in Trás-os-Montes and Beira Baixa and 117 from red deer in Beira Baixa. The seroprevalence data by species and geographic region are presented in Table 1 and Figure 1. These values were defined based on estimates from the Gaussian mixture model.

**Table 1:** Geographic region and exposure results in hunted wild boars and red deer, defined based on the estimates generated by the Gaussian mixture model. Influence of the two regions (Beira Baixa and Trás-os-Montes) on seropositivity of wild boar.

	Number of Animals	Seropositivity n (%)	CI 95% ^a^	*p*-Value OR (95% CI)
Wild Boar	162	11 (6.8)	3.4–11.8	
Trás-os-Montes	89	2 (2.2)	0.3–7.9	0.024 6.06 (1.2–59.47)
Beira Baixa	73	9 (12.3)	5.8–22.1	
Red Deer				
Beira Baixa	117	16 (13.7)	8.0–21.3	-

^a^ CI—Confidence interval.

The positivity threshold was defined as the intersection point of the fitted negative and positive Gaussian distributions, corresponding to the S/P% value at which both components had equal probability density. Samples with S/P% values above this threshold were classified as seropositive, and those below the threshold as seronegative. This intersection yielded an estimated cut-off of approximately 18.2% S/P for wild boar and 13.3% S/P for red deer. In the present study, the overall exposure found for *C. burnetii* in wild boar was 6.8% (11/162; 95% CI: 3.4% to 11.8%). Of these 11 positive wild boars, 2/89 (2.2%) were from the region of Trás-os-Montes and 9/73 (12.3%) from Beira Baixa. Statistically significant differences were observed in the *C. burnetii* seropositivity between the two wild boar regions (*p* < 0.05).

**Figure 2:**
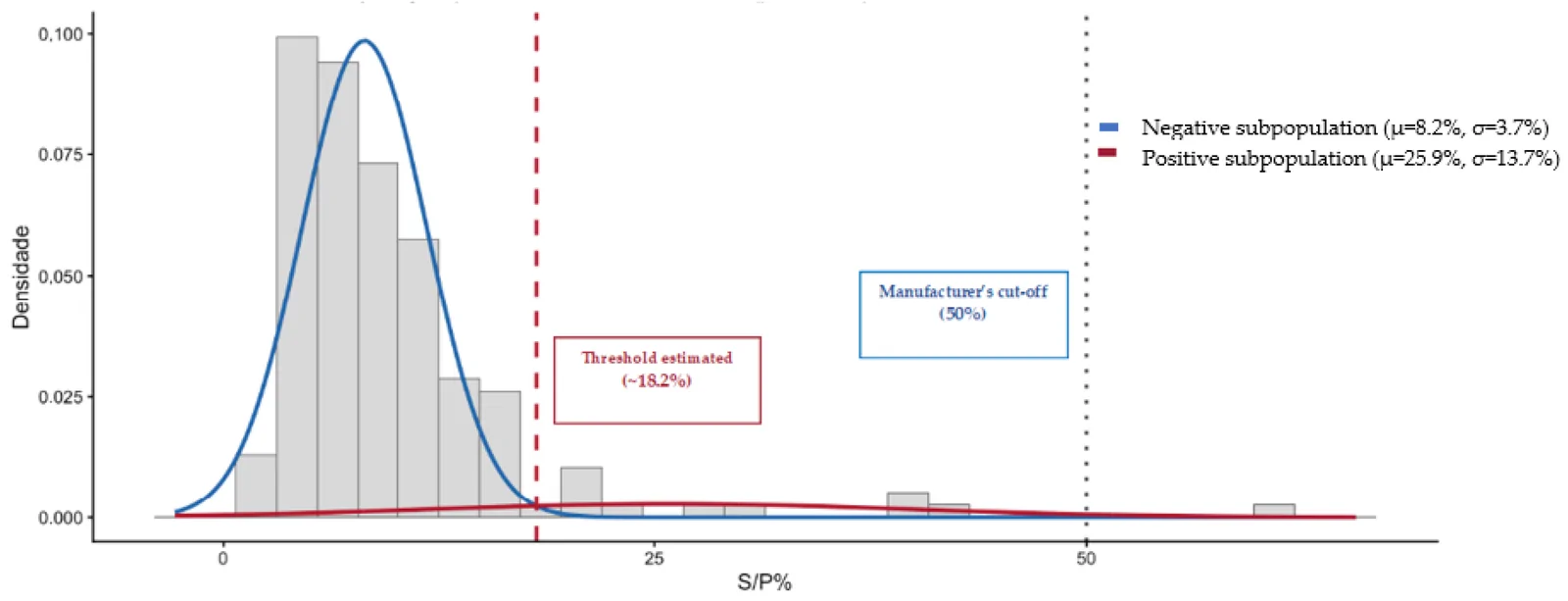
Frequency distribution of observed S/P% values (grey bars) in the modified indirect ELISA for wild boar (*n* = 162). Solid curves correspond to the fitted distributions using the mean and variance of the two distributions from the EM analysis.

**Figure 3:**
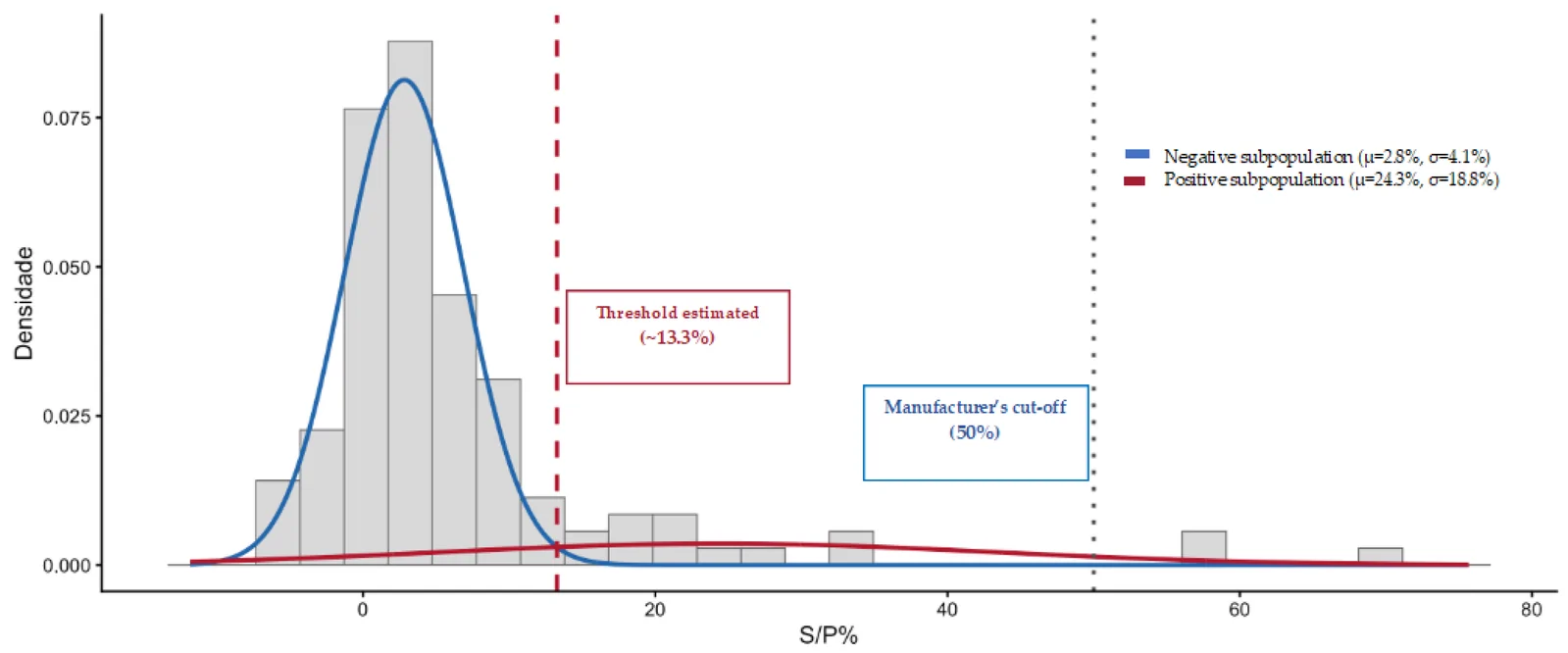
Frequency distribution of observed S/P% values (grey bars) in the modified indirect ELISA for red deer (*n* = 117). Solid curves correspond to the fitted distributions using the mean and variance of the two distributions from the EM analysis.

The EM mixed model converged upon two distinct distributions for wild boar. The mean S/P% was estimated at 8.2% (SD 3.7%) and 25.9% (SD 13.7%), representing the means of the negative and positive wild boar sub-population distributions, respectively. The threshold was set at 18.2%, resulting in an apparent increase in seroprevalence compared with the manufacturer’s 50% cut-off (0.6%; 95% CI: 0.0–3.4%). All the samples were considered based on the estimated S/P% (Figure 2). Concerning the red deer from Beira Baixa, antibodies against *C. burnetii* were found in 16/117 animals (13.7%; 95% CI: 8.0–21.3%). The EM model likewise converged on two distinct distributions for red deer, with mean S/P% values of 2.8% (SD 4.1%) and 24.3% (SD 18.8%) for the negative and positive sub-populations, respectively. The threshold was set at 13.3%, resulting in an apparent increase in seroprevalence compared with the manufacturer’s 50% cut-off (2.6%; 95% CI: 0.5–7.3%). Likewise, all samples were incorporated into the calculation of the S/P% estimate (Figure 3).

The summary of the overall seroprevalence and the agreement between the two classification approaches for both species is presented in Table 2.

After application of the Gaussian mixture model-derived threshold, 10 wild boar samples were reclassified as positive, corresponding to 6.2% of the samples shifting from negative to positive as a result of the lower positivity cut-off, which decreased from 50% to approximately 18.2%. In red deer, 13 of 117 samples (11.1%) were reclassified under the new approach. All of these reclassifications were from negative to positive, indicating that the new method increased the number of positive samples.

Agreement between the two classification methods, assessed by Cohen’s kappa coefficient (κ) with 95% confidence intervals, was 0.285 and 0.15, respectively, for red deer and wild boar.

Between the two hunting species, exposure to red deer (13.7%; 95% CI: 8.0% to 21.3%) was higher than in wild boar (6.8%; 95% CI: 3.4% to 11.8%).

## 4. Discussion

This study reports serological exposure to *Coxiella burnetii* in Portuguese wild ungulates, with 6.8% seropositivity in wild boar and 13.7% in red deer, using an indirect commercial multi-species ELISA and an empirically derived species-specific threshold from a Gaussian mixture model. While these results indicate circulation of the pathogen in east-central and northeastern Portugal, they should be interpreted as evidence of exposure rather than infection or reservoir status. Accordingly, the epidemiological role of these species in the wildlife–livestock–human interface remains to be elucidated.

The higher seroprevalence observed in wild boar (12.3%) and red deer (13.7%) from Beira Baixa may reflect local ecological and management conditions. Possible drivers include wild ungulate density, habitat loss due to urban expansion, declines in natural predator populations, and hunting pressure. However, these factors should be regarded only as speculative and warrant further investigation [26,27].

Although a larger number of wild boars were sampled in Trás-os-Montes (89/162) than in Beira Baixa (73/162), the exposure in Trás-os-Montes was markedly lower (2.2%; 2/89) compared with the higher value recorded in Beira Baixa (12.3%; 9/73). The difference in exposure between the two regions was statistically significant (*p* < 0.05). When the red deer seropositive results (16/117; 13.7%) are also considered, the combined findings suggest that the predominance of extensive domestic ruminant systems in central Portugal, with a high density of these ungulates, may facilitate interspecies host contact, thereby increasing opportunities for *C. burnetii* transmission [41,42,43].

In the study areas, with the ongoing increase in wild boar and red deer populations, hunting activities involving wild ungulates are particularly important, providing conditions for studying *C. burnetii* infection at the wildlife–livestock–human interface [44]. The proximity between humans and domestic ruminants, coupled with the potential spillover of infection to wildlife and the intense hunting activity in Beira Baixa, may strengthen the wildlife–livestock–human interface and thereby increase the risk of infection if not accompanied by appropriate prevention and biosecurity measures [39,45,46,47]. Since all samples were collected from legally hunted wild animals, no data were available for red deer in Trás-os-Montes because hunting pressure on this species is comparatively low. In contrast, wild boars are the predominant game species in the region. In this context, the zoonotic risk associated with hunting activities should be carefully considered [21,48]. These findings may have implications for risk assessment, particularly among hunters who manage wildlife carcasses, and support consideration of personal protective measures and the avoidance of raw or undercooked meat [21].

In Portugal, only one study on wild ungulates had been conducted to date using an indirect multi-species commercial ELISA kit, reporting seroprevalences of 1.1% and 1.9% in wild boar and red deer, respectively [18]. Other recent studies were performed mostly in domestic ruminants [43,49,50], dogs and cats [51], and humans [52,53]. Compared with studies from other countries, in Spain, the overall seroprevalence against *C. burnetii* was 1.5% anti-*C burnetii* antibodies found in 1.8% of mouflon, 1.5% of red deer, and 1.0% of Iberian ibex [54]. In Australia, the overall seroprevalence of *C. burnetii* antibodies in wild deer was 3.4% with true prevalence estimated to be 4.3% [55]. In another study in Spain, among wild ungulates, *C. burnetii* DNA was detected in 7.0% of roe deer, 1.9% of wild boar and 2.4% of red deer [56].

However, comparison of data may not be feasible. The studies were conducted in various geographical areas, using different methods, and changes in the epidemiology throughout the years might have occurred. From a methodological perspective, our results highlight key challenges in interpreting serological data for *C. burnetii* in wildlife. The commercial multi-species ELISA used was developed and validated for domestic ruminants, and there are no species-specific reference sera or validated cut-offs for wild boar and red deer. Using the manufacturer’s recommended cut-off (>50% S/P) resulted in lower seroprevalence estimates, particularly in wild boar (0.6%). In contrast, the empirical, species-specific cut-offs derived from Gaussian mixture models changed the proportion of samples classified as positive, yielding 6.8% in wild boar and 13.7% in red deer. The slight to fair agreement between the two approaches (κ = 0.285 in red deer; κ = 0.15 in wild boar) suggests that, while the manufacturer’s cut-off provides a conservative baseline, data-driven thresholds may offer a more nuanced classification when interpreted with caution. Cohen’s kappa coefficient was used to assess the agreement between the classifications obtained from the Gaussian mixture model (GMM) and the reference ELISA interpretation based on the manufacturer’s cut-off. The estimated values were κ = 0.15 for wild boar and κ = 0.285 for red deer, corresponding, respectively, to slight (0.00–0.20) and fair (0.21–0.40) agreement according to the Landis and Koch classification. In fact, the wide confidence intervals (Table 2), particularly for wild boar, indicate considerable uncertainty in these estimates, reflecting the limited number of seropositive animals and the overlap between the seronegative and seropositive subpopulations. Rather than indicating that the GMM is unreliable, this limited agreement is consistent with the manufacturer’s cut-off developed and validated for domestic ruminants, being poorly suited to wild boar and red deer. Under the 50% cut-off, almost no animals were classified as seropositive (0.6% in wild boar and 2.6% in red deer), whereas the species-specific thresholds identified a subpopulation of seroreactive animals that the fixed cut-off failed to capture. The two approaches therefore diverge precisely where a wildlife-adapted interpretation would be expected to differ from a criterion transferred from domestic hosts. Accordingly, the GMM seems to be appropriate as a complementary, data-driven approach for threshold determination, to be interpreted alongside biological and diagnostic considerations rather than as a direct substitute for a validated reference.

The use of mixture models nonetheless has limitations, including the assumption of an underlying bimodal distribution and the need for sufficiently large samples to reliably separate the seronegative and seropositive components. In red deer, the seropositive component was small and comparatively broad (SD = 18.8% S/P), overlapping substantially with the seronegative distribution, which increases the uncertainty in threshold placement and may contribute to the discrepancy between the manufacturer-based and empirical seroprevalence estimates. The lack of standardized control sera and of a validated serological approach for wild species make the interpretation of results and the comparison across studies difficult, and may compromise the reliability of wildlife monitoring as well as the identification of factors influencing *C. burnetii* seroprevalence. These limitations reinforce the need to develop and validate wildlife-specific serological tools and interpretative criteria [39]. Q fever may be considered a zoonotic disease that benefits from integrated collaboration between human and veterinary health sectors within a One Health framework to help mitigate infection risks in both populations [18]. A key challenge will be to reduce human exposure to this zoonosis through a preventive ‘One Health’ approach [57,58]. By providing baseline data on *C. burnetii* exposure in wild boar and red deer in areas where ruminant farming and human activities overlap, this study helps strengthen disease prevention. It supports the development of integrated surveillance systems capable of safeguarding public health. Further investigation into the sources of *C*. *burnetii* infection and the persistence of environmental contamination will be needed in the coming years.

## 5. Conclusions

This study provides serological evidence of exposure to *Coxiella burnetii* in free-ranging wild boar and red deer in central and northeastern Portugal. While these findings support the inclusion of wildlife in *C. burnetii* surveillance programs within a One Health framework, they do not demonstrate that either species contributes to pathogen maintenance in the ecosystem or to transmission to livestock or humans. Further research is needed to clarify the epidemiological role of wildlife and its relevance to Q fever at the wildlife–livestock–human interface.

## Figures and Tables

**Table 2:** Summary of the overall exposure and the agreement between the two classification approaches for wild boar and red deer.

	Results	
	Red Deer	Wild Boar
Seroprevalence (cut-off 50%)		
Number of animals	117	162
Positives—cut-off 50%	3 (2.6%) CI 95: 0.5–7.3%	1 (0.6%) CI 95: 0.0–3.4%
Gaussian Mixture Model		
Estimated threshold (EM model)	~13.3%	~18.2%
Positives—Estimated threshold	16 (13.7%) CI 95: 8.0–21.3%	11 (6.8%) CI 95%: 3.4–11.8%
Agreement between methods		
Kappa Coefficient	0.285 (CI 95%: 0.029–0.541)	0.15 (CI 95%: −0.115–0.429)

## Data Availability

The data presented in this study are available on request from the corresponding author.

## Supplementary Materials

The following supporting information can be downloaded at https://www.mdpi.com/article/10.3390/ani16182876/s1. Text S1: Derivation of species-specific ELISA cut-off values using a two-component Gaussian mixture model; Table S1: Positive (PC) and negative (NC) control optical densities of each ELISA plate, and kit validation criteria. Table S2: Parameters of the two-component Gaussian mixture models fitted to the S/P% distributions of wild boar and red deer, and derived species-specific cut-off values.
