# Supplementary material for: Wild Boar and Red Deer as Key Players in Coxiella Burnetii Circulation in the Wildlife–Livestock–Human Interface: Serological Analysis Using a Gaussian Mixture Model
Source: Animals (Basel). 2026 Sep 12;16(18):2876. doi: 10.3390/ani16182876 (PMC13603785; doi:10.3390/ani16182876)
Supplement: Supplementary file 1 [file animals-16-02876-s001.zip › animals-4439814-supplementary.pdf]

Supplementary Materials S1:

Determination of species-specific ELISA cut-off values using two-component Gaussian mixture models

S1.1. Calculation of S/P% values

For each sample, the S/P% value was calculated with the positive (PC) and negative (NC) control optical densities (OD) of the corresponding ELISA plate, according to the manufacturer's formula:

$$S/P\% = (OD\_sample - OD\_NC) / (OD\_PC - OD\_NC) \times 100$$

All plates fulfilled the kit validation criteria (OD_PC > 0.350 and OD_PC/OD_NC > 3), confirming the technical validity of the assay across runs (Table S1).

Table S1. Positive (PC) and negative (NC) control optical densities of each ELISA plate, and kit validation criteria.

Species	Plate	n	OD PC	OD NC	PC/NC	Validation
Wild boar	1	89	1.6825	0.0550	30.6	Met
Wild boar	2	73	1.6050	0.0415	38.7	Met
Red deer	1	61	1.8385	0.2035	9.0	Met
Red deer	2	56	1.7405	0.2260	7.7	Met

The negative control optical densities differed markedly between species: 0.042 and 0.055 on the two wild boar plates, against 0.204 and 0.226 on the two red deer plates. Because half of the red deer sample optical densities lie between 0.23 and 0.34 (interquartile range), subtraction of the comparatively high NC value leaves only a small residual signal, which accounts for the low S/P% values observed in this species. This is a genuine plate-specific characteristic rather than a computational artefact.

S1.2. Model specification and estimation

For each species separately, the distribution of S/P% values (x) was modelled as a two-component Gaussian mixture:

$$\mathbf{f(x) = \lambda_1 \cdot \varphi(x; \mu_1, \sigma_1) + \lambda_2 \cdot \varphi(x; \mu_2, \sigma_2)}$$

where $\varphi(x; \mu_k, \sigma_k)$ is the normal probability density function of component k (component 1: seronegative subpopulation; component 2: seropositive subpopulation), μ_k and σ_k are the component mean and standard deviation, and λ_1 and λ_2 ($\lambda_1 + \lambda_2 = 1$) are the mixing proportions, i.e., the estimated proportions of seronegative and seropositive animals in the sample.

The five free model parameters (λ_1, μ_1, σ_1, μ_2, σ_2) were estimated by maximum likelihood using the Expectation–Maximisation (EM) algorithm, as implemented in the `normalmixEM` function of the *mixtools* package (Benaglia et al., 2009) in R. Because the EM algorithm converges to local maxima of the likelihood, the model was fitted from 200 random initialisations and the solution with the highest log-likelihood was retained. The stability of the resulting threshold was additionally verified over 50 further random initialisations per species. Model adequacy was assessed by visual inspection of the fitted component densities overlaid on the observed S/P% frequency distributions (Figures 2 and 3 of the main text).

S1.3. Cut-off criterion

The species-specific cut-off c was defined as the S/P% value at which an observation is equally likely to belong to either subpopulation, i.e., the value at which the posterior probability of membership in the seropositive component equals 0.5. Under the fitted model, this corresponds to the intersection point of the two *weighted* component densities:

$$\boldsymbol{\lambda_1 \cdot \varphi(c; \mu_1, \sigma_1) = \lambda_2 \cdot \varphi(c; \mu_2, \sigma_2)}$$

Samples with S/P% above c have a posterior probability greater than 0.5 of belonging to the seropositive subpopulation and were classified as seropositive; samples below c were classified as seronegative.

Note that this criterion does not correspond to the midpoint between the two component means. Because the mixing proportions are unequal ($\lambda_1 > \lambda_2$ in both species) and the component standard deviations differ, the equal-posterior-probability point is displaced relative to the midpoint, and the direction of that displacement depends on the balance between the two effects. In wild boar, where the two components have comparatively similar dispersions, the larger weight of the seronegative component displaces the threshold above the midpoint (18.2% versus a midpoint of 17.1%). In red deer, where the seropositive component is far more dispersed (σ_2 = 18.8% against σ_1 = 4.1%), this effect is offset and the threshold falls slightly below the midpoint (13.3% versus 13.6%). The root of the equation above lying between μ_1 and μ_2 was obtained numerically (uniroot function in R).

S1.4. Estimated parameters

Table S2. Parameters of the two-component Gaussian mixture models fitted to the S/P% distributions of wild boar and red deer, and derived species-specific cut-off values.

Parameter	Wild boar (n = 162)	Red deer (n = 117)
Seronegative component — mixing proportion, λ_1	0.904	0.830
Seronegative component — mean, μ_1 (S/P%)	8.2	2.8
Seronegative component — SD, σ_1 (S/P%)	3.7	4.1
Seropositive component — mixing proportion, λ_2	0.096	0.170
Seropositive component — mean, μ_2 (S/P%)	25.9	24.3
Seropositive component — SD, σ_2 (S/P%)	13.7	18.8
Cut-off (equal posterior probability), c (S/P%)	18.2	13.3

The threshold was stable across random initialisations in both species. In red deer, the overlap between the two fitted components is considerable; the empirical threshold should therefore be interpreted as a complementary, data-driven criterion rather than a validated diagnostic cut-off (see Discussion of the main text).

S1.5. R code

The analysis can be reproduced with the following R code, where `sp` is the numeric vector of S/P% values for a given species:

```
library(mixtools)
set.seed(20260803)

# Fit the two-component mixture from 200 random initialisations and
# keep the solution with the highest log-likelihood
best <- NULL
for (k in 1:200) {
  fit <- try(normalmixEM(sp, k = 2, maxit = 5000, epsilon = 1e-10,
                         verb = FALSE), silent = TRUE)
  if (inherits(fit, "try-error")) next
  if (is.null(best) || fit$loglik > best$loglik) best <- fit
}

# Order components so that component 1 is the seronegative (lower-mean) one
o      <- order(best$mu)
mu     <- best$mu[o]       # component means
sigma  <- best$sigma[o]    # component standard deviations
lambda <- best$lambda[o]   # mixing proportions

# Cut-off: intersection of the weighted component densities
# (equal posterior probability of membership in either component)
g <- function(x) {
  lambda[1] * dnorm(x, mu[1], sigma[1]) - lambda[2] * dnorm(x, mu[2], sigma[2])
}
cutoff <- uniroot(g, interval = c(mu[1], mu[2]), tol = 1e-10)$root
cutoff
```
